# Comparison of local tumor control in patients with HCC treated with SBRT or TACE: a propensity score analysis

**DOI:** 10.1186/s12885-018-4696-8

**Published:** 2018-08-09

**Authors:** Dominik Bettinger, Eleni Gkika, Michael Schultheiss, Nicolas Glaser, Sophie Lange, Lars Maruschke, Nico Buettner, Simon Kirste, Ursula Nestle, Anca-Ligia Grosu, Robert Thimme, Thomas B. Brunner

**Affiliations:** 1 Department of Medicine II, Medical Center University of Freiburg, Faculty of Medicine, University of Freiburg, Hugstetter Str. 55, D-79106 Freiburg, Germany; 2 Berta-Ottenstein-Programme, Faculty of Medicine, University of Freiburg, Freiburg, Germany; 3 Department of Radiation Oncology, Medical Center University of Freiburg, Faculty of Medicine, University of Freiburg, Robert-Koch-Str. 3, D-79106 Freiburg, Germany; 4 Department of Radiology, Medical Center University Freiburg, Faculty of Medicine, University of Freiburg, Hugstetter Str. 55, D-79106 Freiburg, Germany; 5 Department of Radiation Oncology, Kliniken Maria Hilf, Moenchengladbach, Germany; 6 German Cancer Consortium (DKTK), Partner Site Freiburg, Freiburg, Germany; 7 German cancer Research Center (DKFZ), Heidelberg, Germany; 8 Department of Radiotherapy, University of Magdeburg, Magdeburg, Germany

**Keywords:** Hepatocellular carcinoma, Transarterial chemoembolization, Stereotactic body radiation therapy, Propensity score analysis, Overall survival

## Abstract

**Background:**

As stereotactic body radiation therapy (SBRT) has shown to be effective and safe in patients with hepatocellular carcinoma (HCC), the aim of our propensity score matched analysis was to evaluate the efficacy of SBRT in comparison to transarterial chemoembolization (TACE) in intermediate and advanced HCC.

**Methods:**

Patients treated with TACE (*n* = 367) and patients allocated to SBRT (*n* = 35) were enrolled in this study. Propensity score matching was performed to adjust for differences in baseline and tumor characteristics of TACE and SBRT patients. Local tumor control (LC) 1 year after treatment, overall survival (OS) and 1-year mortality were assessed.

**Results:**

Patients treated with SBRT have received more prior HCC treatments compared to TACE patients. The LC 1 year after treatment in the unmatched cohort was 74.4% for TACE patients compared to 84.8% in the SBRT group. Patients treated with TACE showed significantly improved OS (17.0 months vs. 9.0 months, *p* = 0.016). After propensity score matching, the LC in the TACE (*n* = 70) and SBRT (*n* = 35) group was comparable (82.9% vs. 84.8%, *p* = 0.805) and OS did not differ significantly in both groups.

**Conclusions:**

SBRT after prior HCC therapy in selected patients shows comparable LC at 1 year, OS and 1-year mortality compared to patients treated with TACE.

**Electronic supplementary material:**

The online version of this article (10.1186/s12885-018-4696-8) contains supplementary material, which is available to authorized users.

## Background

Hepatocellular carcinoma (HCC) is often diagnosed in intermediate or advanced tumor stages and treatment options are limited [1, 2]. According to the Barcelona Clinic Liver Cancer (BCLC) classification [1, 3], patients with intermediate HCC (BCLC B) are treated with transarterial chemoembolisation (TACE) [4] and there is growing evidence that patients with BCLC C without complete portal vein thrombosis (PVT) and even with extrahepatic metastases may also benefit from TACE [5].

During the last years, stereotactic body radiation therapy (SBRT) has emerged as another local ablative non-invasive treatment approach in patients with HCC [6–8]. It has been reported that SBRT can achieve high rates of local tumor control with acceptable toxicity in patients with HCC, also in carefully selected patients with impaired liver function [6, 9]. Although these reports have shown that SBRT is a feasible and well-tolerated treatment option for patients with HCC, there is no consensus in which setting SBRT should be used. SBRT was also used to bridge to liver transplantation as an alternative treatment option to TACE with favorable results [10–12]. However, there are no studies evaluating the efficacy of SBRT compared to TACE in patients with intermediate HCC outside the transplantation setting. In order to analyze this important clinical issue, we performed a single-center, retrospective analysis by using propensity score matching focusing on local tumor control, overall survival (OS) and 1-year-mortality.

## Methods

### Selection of patients

The TACE cohort consisted of patients who had been treated at the University Hospital Freiburg (Germany) between January 2003 and January 2015. In summary, 1030 HCC patients were included in an HCC database. Of these patients, 407 were initially treated with TACE. Patients with extrahepatic metastases who had been treated by TACE in an individual treatment approach were excluded from this analysis. Further, we excluded patients with BCLC A, who received TACE as a bridge to surgery or liver transplantation. In summary, 367 patients who have been treated with TACE were included in these analyses.

The SBRT cohort consisted of 35 consecutive patients with 49 HCC lesions who have been treated in the Department of Radiation Oncology of the University Hospital Freiburg (Germany) between 2012 and 2016 and which have partly been published elsewhere [6, 13]. Patients treated with SBRT who received prior TACE were not included in the TACE group. Treatment decisions were made at the dedicated institutional multidisciplinary HCC tumor board following institutional, national and international guidelines. Typically, TACE was the first-line treatment in patients without complete portal vein thrombosis. SBRT was performed after TACE failure, as an alternative to systemic treatment with sorafenib or after progression during sorafenib. Therefore, these patients have mainly received prior HCC therapy.

### Definitions

HCC was staged using the Barcelona Clinic Liver Cancer (BCLC) classification. Diagnosis of HCC was made according the current guidelines mainly by imaging (computer tomography [CT] or dynamic contrast-enhanced magnetic resonance imaging [MRI]) when lesions showed the typical arterial phase hyperenhancement and portal venous and/or delayed washout [1, 3]. The number of focal hepatic lesions and the maximum diameter as well as the presence of portal vein thrombosis (PVT) were assessed. We summarized the intrahepatic lesions in oligonodular (one or two intrahepatic lesions) and in multifocal HCC (three or more lesions or diffuse HCC growth pattern).

### TACE procedure

TACE was performed using a selective or super-selective approach. Intra-arterial infusion of the chemotherapeutic agent and lipiodol was performed after having localized the target lesion. Epirubicin or mitomycin were used as chemotherapeutic agents. The chemotherapeutic agent was not defined in the study protocol. The lipiodol infusion was stopped when intra-arterial stasis was observed in the angiographic control. Further, gelatin sponge particles or PVA particles were used for embolization. In 41 patients (11.2%) drug-eluting beads TACE (DEB-TACE) was performed.

### SBRT techniques

In order to exactly define the radiation field, patients were immobilized in supine position with a vacuum cushion (BlueBAG BodyFIX, Innovative Technologies Völp, Innsbruck, Austria) and underwent 4 dimensional-CT (4D CT, Brilliance CT Big Bore, Philips Medical Systems, Cleveland, OH) as previously described [6, 13]. For the 4D acquisition (Mayo Clinic Respiratory feedback system), we monitored breathing which was reduced with an abdominal compression method. Lesions with contrast enhancement in the arterial phase and with washout in the venous phase and/or delayed phase including the portal vein thrombosis (PVT) were defined as gross tumor volume (GTV). The internal target volume (ITV) was created to account for the extent and the position of the tumor at all motion phases of the 4D-CT data set, and the PTV a uniform expansion of 4 mm of the ITV. Further, for using image guide radiotherapy (IGRT) lipiodol deposits from previous TACE sessions were used. In the absence of lipiodol as a marker, fiducial markers were implanted before beginning of radiotherapy. The decision for the numbers of fractions which were delivered to the patients was based on the proximity to organs at risk such as the stomach, the small intestine and the colon: In patients without a close proximity to these critical structures 3 fractions (3 × 12.5–15 Gy) were preferred. In contrast, 12 fractions (12 × 4–5.5 Gy) were applied if there was a close contact to the OARs and 5 fractions (5 × 7–10 Gy) were used in case of intermediate closeness to the OARs. On every treatment day, before starting radiation therapy, a cone beam computed tomography (CBCT) with oral contrast for visualizing the stomach and/or the duodenum was performed. Therefore, according to the current location of the OARs, corrections in the radiation fields were done on each treatment day if necessary.

In some lesions, dose constraints could not be achieved. In these patients, we used a simultaneous integrated protection (SIP) dose prescription without reducing the dose to the entire PVT [14]. During the study period, treatments were either prescribed to the 60% and 80% encompassing isodose (between 2007 and 2013) or according to ICRU report 83 (after 2013). The prescribed doses were converted to equieffective doses for 2 Gy fractions (EQD2) using an α/β ratio of 10 Gy and 3 Gy to account for tumour and late reacting bowel tissue, respectively.

### Radiological assessment

Radiological response was assessed every 3 months after TACE or SBRT by using the mRECIST criteria (version 1.1) [13]. Complete remission, partial remission or stable disease was summarized as local tumor control (LC). Patients treated with TACE with detection of residual HCC within the target lesion during follow-up imaging were allocated to further TACE sessions. Concerning the LC at 1 year in TACE patients we included the target lesions and reported the response assessment at 1 year. Patients who received more than one TACE session due to residual tumor disease in the target lesion were *not* classified as non-responders.

For response assessment in patients treated with SBRT, imaging was reviewed by comparing the treatment plan for SBRT. By using this approach, we were able to define if there was local recurrence or a new untreated tumor. The LC of SBRT patients was assessed considering all treated lesions (*n* = 49).

### Statistical analyses

The present study was a retrospective observational study. Baseline characteristics of the patients were analyzed before TACE or SBRT. The primary outcome in our analysis was LC 1 year after treatment and the secondary outcome were overall survival (OS), 1-year-mortality and toxicity. Continuous variables are reported as mean with standard deviation whereas categorical variables are expressed as frequencies and percentages (in parentheses) unless stated otherwise. For continuous variables, differences were determined using Wilcoxon-Mann-Whitney and Kruskal-Wallis tests. We used non-parametric tests as there was no Gaussian distribution of the data which was confirmed by the Kolmogorov-Smirnov test before starting the analyses. χ^2^ tests or Fisher’s Exact tests were used for categorical variables. *P* values < 0.05 were considered being significant.

Overall survival was defined from the day prior to TACE or SBRT until death or last follow-up. At the end of the observation period (01/07/2017) 358 patients (89.1%) in the whole cohort and 86 patients (81.9%) in the matched cohort had died. Survival was calculated using Kaplan-Meier analyses. Differences in survival were assessed using logRank tests.

As the outcome parameters may be influenced by patient selection for either TACE or SBRT, we performed propensity score matching. For development of the propensity score, we performed multivariable logistic regression model including the following parameters: ECOG 0 vs. 1/2, segmental portal vein thrombosis (PVT), hepatic tumor expansion (oligonodular vs. multifocal), tumor size, Child score and viral liver disease. Due to the large differences of the frequency of previous treatment between the treatment groups, we were not able to adjust for this bias, as this would have resulted in very small numbers in each group after propensity score matching. After the propensity score has been established, we preformed 2:1 matching. For matching we used the nearest-neighbour matching method with a calliper with of 0.01 without replacement. Standardized differences were calculated in order to assess post-hoc balance [15]. The standardized differences before and after matching are presented in the supplementary file.

Statistical analyses were performed with SPSS (version 24.0, IBM, New York, USA) and GraphPad Prism (version 6, GraphPad Software, San Diego, CA, USA).

## Results

### Patient characteristics

Baseline characteristics are summarized in Table 1. In the TACE cohort there were significantly more patients with viral liver disease compared to the SBRT cohort (31.1% vs. 11.4%, *p* = 0.018). Patients treated with SBRT presented with more advanced tumor disease compared to patients with TACE as they were more often classified as BCLC C (18.5% vs. 31.4%, *p* = 0.046). 60.8% of the patients treated with TACE had multifocal HCC compared to 83.0% of the patients in the SBRT group (*p* = 0.010). Only 5 patients (1.3%) in the TACE group had been treated before study inclusion compared to 83.0% in the SBRT group (*p* < 0.001). SBRT patients presented with a higher Child score compared to TACE patients (5.9 ± 1.3 vs.8.4 ± 7.1, *p* = 0.001). Technical data of SBRT are summarized in Table 1.

**Table 1:** Baseline characteristics of study patients and lesions treated

Characteristics	TACE	SBRT	*p* value
	*n* = 367	*n* = 35	
Gender			0.802
Male	314 (85.6)	29 (83)	
Female	53 (14.4)	6 (17)	
Age in years	66.8 ± 9.2	69.0 ± 8.1	0.305
ECOG^1^			
0	277 (75.5)	23 (65.7)	0.224
1	43 (11.7)	12 (34.3)	0.001
2	47 (12.8)	0	0.023
Etiology of liver disease			0.018
Viral	114 (31.1)	4 (11.4)	
Non-viral	253 (68.9)	31 (88.6)	
Child Score	5.9 ± 1.3	6.4 ± 1.3	0.006
Child A	269 (73.3)	19 (4.3)	0.020
Child B	95 (25.9)	16 (45.7)	0.017
Child C	3 (0.8)	0	0.999
Previous treatment^a^	5 (1.3)	29 (83.0)	< 0.001
None	362 (98.6)	6 (17.1)	< 0.001
Surgery	2 (0.5)	8 (22.9)	0.899
Sorafenib	2 (0.5)	1(2.9)	0.324
TACE	1 (0.3)	28(80.0)	< 0.001
Intrahepatic tumor expansion			0.010
Oligonodular	144 (39.2)	6 (17)	
Multifocal	223 (60.8)	29 (83)	
BCLC^2^			0.046
B	299 (81.5)	24 (68.6)	
C	68 (18.5)	11 (31.4)	
Largest tumor diameter [cm]	6.1 ± 3.4	8.4 ± 7.1	0.001
Segmental PVT^4^	68 (18.5)	11 (31.4)	0.076
Laboratory			
Platelets [10^3^/μl]	187 ± 115	183 ± 131	0.263
AST^7^ [U/l]	90 ± 80	99 ± 66	0.238
ALT^8^ [U/l]	66 ± 58	55 ± 40	0.335
Bilirubin [mg/dl]	1.2 ± 1.2	1.8 ± 1.8	0.656
Albumin [g/dl]	3.6 ± 0.6	3.4 ± 0.5	0.034
AFP^15^ [ng/ml]	4792.4 ± 25,171.7	2279.8 ± 9386.5	0.493
Technical data TACE^3^ and SBRT^5^			
TACE			
cTACE^6^	326 (88.8)		
Drug-eluting beads TACE	41 (11.2)		
Number of TACE sessions	2 ± 1		
Two TACE	253 (68.9)		
Three TACE	84 (22.9)		
Four TACE	30 (8.2)		
SBRT		median (IQR^14^)	
Total prescribed dose (TD)		45 (42–50) Gy	
EQD2_10,TD_^9^		56 (54–83) Gy	
D_max_^10^		53 (50–57) Gy	
EQD2_10,Dmax_^11^		82 (62–98) Gy	
D_mean,liver_^12^		17 (14–25) Gy	
EQD2_Dmean,liver_^13^		20 (14–36) Gy	

^a^Patients treated with SBRT have received more than one treatment

*Abbreviations:* ^*1*^*ECOG* Eastern Cooperative Oncology Group, ^*2*^*BCLC* Barcelona Clinic Liver Cancer ^*3*^*TACE* transarterial chemoembolization, ^*4*^*PVT* portal vein thrombosis*,* ^*5*^*SBRT* stereotactic body radiation therapy*,* ^*6*^*cTACE* conventional transarterial chemoembolization*,* ^*7*^*AST aspartat aminotransferase,* ^*8*^*ALT alanine aminotransferase,* ^9^*EQD2*_*10,TD*_ equieffective doses for 2 Gy fractions of the prescribed dose, ^*10*^*D*_*max*_ Maximum point dose, ^*11*^*EQD2*_*10,Dmax*_ equieffective doses for 2 Gy fractions of the maximum point dose, ^*12*^*D*_*mean,liver*_ Mean liver dose, ^*13*^*EQD2*_*Dmean,liver*_ equieffective doses for 2 Gy fractions of the mean liver dose, ^*14*^*IQR* interquartile range, ^*15*^*AFP* alpha-fetoprotein

### Local tumor control at 1 year, OS and 1-year mortality in patients treated with TACE or SBRT

**Fig. 1:**
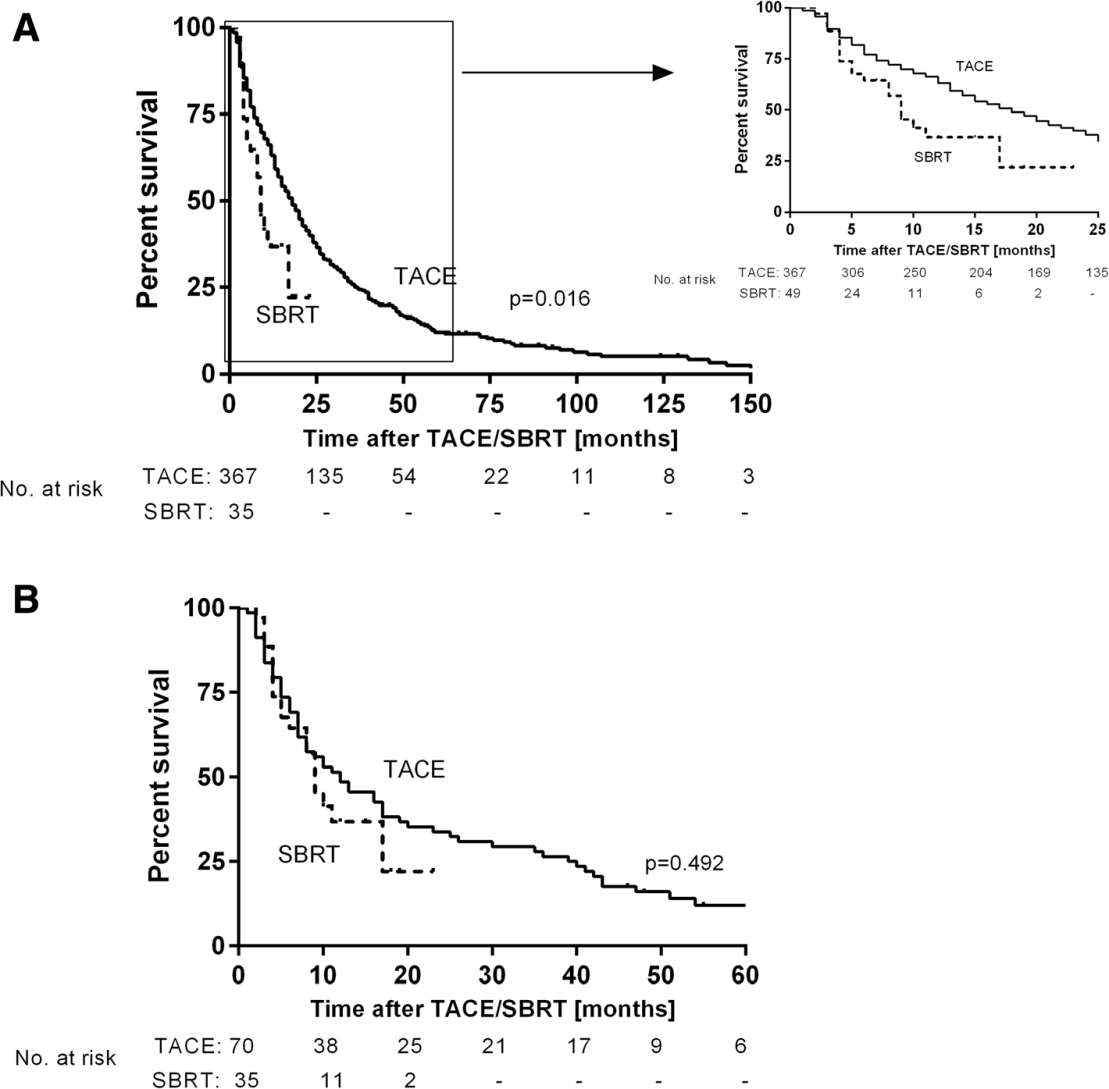
Overall survival in patients with transarterial chemoembolization and SBRT in the unmatched (a) and matched cohort (b)

In patients treated with TACE the LC at 1 year was 74.4% compared to 84.8% in patients treated with SBRT (*p* = 0.146). There was a trend to a better LC in patients treated with SBRT (Table 3). Patients with TACE had a median OS of 17.0 [14.4–19.6] months compared to 9.0 [6.7–11.3] months in SBRT patients (*p* = 0.016) (Fig. 1a). 1-year-mortality was higher in patients treated with SBRT compared to TACE patients but did not reach statistical significance (38.4% vs. 53.1%, *p* = 0.073, Table 3).

### Toxicity

The most common toxicity in patients treated with TACE was abdominal pain (*n* = 118, 32.2%), fever (*n* = 84, 22.9%) and nausea and vomiting (*n* = 51, 14.0%). These complications developed shortly after TACE and were explained by a postembolization syndrome. Thirteen patients (3.5%) developed hematoma after puncture of the femoral artery for angiography during the TACE procedure. Three patients (0.8%) developed liver abscess after TACE which was treated by insertion of a percutaneous drain and antibiotic treatment.

The adverse events of the SBRT patients included in this study have partly been published in previous studies [6, 13]. Three of these patients developed gastric ulcer bleeding, three, four and 5 months after treatment. These patients were treated with proton pump inhibitors (2 patients, CTC grade 2) and transfusion (1 patient, grade CTC 3). Importantly, the patient who developed CTC grade 3 gastroduodenitis had previously been treated with SBRT for another HCC lesion 4 months ago. Liver-associated toxicity with a deterioration of liver function assessed by an increase of the Child score was observed in 4 patients mainly with a small increase of the Child score (Child B7 to B8 and Child A6 to B7, Child A5 to A6). Only one patients showed an increase of two points of the Child score (Child A6 to B8) which was attributed to RILD. But this patient fully recovered from this deterioration of liver function and died 9 months after SBRT due to renal failure which was not attributed to treatment. The patient with an increase of the Child score from A5 to A6 after SBRT developed further hepatic decompensation without HCC progression and died 4 months after SBRT. One patient developed a necrotic abscess of the liver due to a dislocation of an indwelling Pigtail-catheter of the bile duct after stent-exchange which was surgically managed and was not related to the SBRT.

### Propensity score matching

As treatment allocation for TACE or SBRT is biased due to different patient and tumor characteristics, we performed propensity score matching to adjust for the imbalance concerning these factors. Multivariable logistic regression (Additional file 1: Table S1) analysis was performed and 105 patients after 2:1 matching (70 patients in the TACE group and 35 patients in the SBRT group) with comparable patients and tumor characteristics were identified (Table 2).

**Table 2:** Baseline characteristics of patients and treated lesions after propensity score matching

Characteristics	TACE	SBRT	*p* value
	*n* = 70	*n* = 35	
Gender			0.543
Male	62 (88.6)	29 (83.0)	
Female	8 (11.4)	6 (17.0)	
Age in years	66.8 ± 9.9	69.0 ± 8.1	0.514
ECOG^1^			0.999
0	45 (64.3)	23 (65.7)	
1/2	25 (35.7)	12 (34.3)	
Etiology of liver disease			0.999
Viral	8 (11.4)	4 (11.4)	
Non-viral	62 (86.6)	31 (88.6)	
Child Score	6.4 ± 1.5	6.4 ± 1.3	0.952
Child A	40 (57.1)	19 (4.3)	0.836
Child B	30 (81.4)	16 (45.7)	0.836
Previous treatment^a^	2 (2.9)	29 (83.0)	< 0.001
None	68 (97.1)	6 (17.1)	< 0.001
Surgery	1 (1.4)	8 (22.9)	0.879
Sorafenib	1 (1.4)	1 (2.9)	0.001
TACE	0	28 (80.0)	< 0.001
Intrahepatic tumor expansion			0.999
Oligonodular	13 (18.6)	6 (17)	
Multifocal	57 (81.4)	29 (83)	
BCLC^2^			0.999
B	49 (70.0)	24 (68.6)	
C	21 (30.0)	11 (31.4)	
Largest tumor diameter [cm]	8.3 ± 4.1	8.4 ± 3.9	0.845
Segmental PVT^4^	21 (30.0)	11 (31.4)	0.999
Laboratory			
Platelets [10^3^/μl]	211 ± 157	183 ± 131	0.217
AST^7^ [U/l]	98 ± 83	99 ± 66	0.742
ALT^8^ [U/l]	69 ± 61	55 ± 40	0.280
Bilirubin [mg/dl]	1.6 ± 1.5	1.8 ± 1.8	0.511
Albumin [g/dl]	3.6 ± 0.7	3.4 ± 0.5	0.109
AFP^15^ [ng/ml]	3255.4 ± 10,907.7	2279.8 ± 9386.5	0.435
Technical data TACE^3^ and SBRT^5^			
TACE			
cTACE^6^	70 (100.0)		
Drug-eluting beads TACE	0		
Number of TACE sessions	2 ± 1		
Two TACE	49 (70.0)		
Three TACE	21 (30.0)		
SBRT		median (IQR^14^)	
Total prescribed dose (TD)		45 (42–50) Gy	
EQD2_10,TD_^9^		56 (54–83) Gy	
D_max_^10^		53 (50–57) Gy	
EQD2_10,Dmax_^11^		82 (62–98) Gy	
D_mean,liver_^12^		17 (14–25) Gy	
EQD2_Dmean,liver_^13^		20 (14–36) Gy	

^a^Patients treated with SBRT have received more than one treatment

*Abbreviations:* ^*1*^*ECOG* Eastern Cooperative Oncology Group*,* ^*2*^*BCLC* Barcelona Clinic Liver Cancer*,* ^*3*^*TACE* transarterial chemoembolization*,* ^4^PVT *portal vein thrombosis,* ^*5*^*SBRT* stereotactic body radiation therapy*,* ^*6*^*cTACE* conventional transarterial chemoembolization*,* ^*7*^*AST* aspartat aminotransferase*,* ^*8*^*ALT* alanine aminotransferase*,* ^9^*EQD2*_*10,TD*_ equieffective doses for 2 Gy fractions of the prescribed dose, ^*10*^*D*_*max*_ Maximum point dose, ^*11*^*EQD2*_*10,Dmax*_ equieffective doses for 2 Gy fractions of the maximum point dose, ^*12*^*D*_*mean,liver*_ Mean liver dose, ^*13*^*EQD2*_*Dmean,liver*_ equieffective doses for 2 Gy fractions of the mean liver dose, ^*14*^*IQR* interquartile range, ^*15*^*AFP* alpha-fetoprotein

In the matched cohort, the LC at 1 year in the TACE group was 82.9% compared to 84.8% (*p* = 0.805, Table 3). With regards to the OS in both cohorts, patients treated with TACE had similar OS compared to patients treated with SBRT (11.0 [5.9–16.1] months for TACE patients vs. 9.0 [6.7–11.3] months in SBRT patients, *p* = 0.492, Fig. 1b). 1-year-mortality was 52.9% in the TACE cohort compared to 53.1% in the SBRT group (*p* = 0.989, Table 3).

**Table 3:** Summary of local tumor control and 1-year mortality in the unmatched and matched cohort in all patients and stratified in BCLC B and C

	Unmatched cohort			Matched cohort		
	TACE	SBRT	*p* value	TACE	SBRT	*p* value
All patients	*n* = 367	*n* = 35		*n* = 70	*n* = 35	
Target lesions	367	46		70	46	
Local tumor control^b^ *n* (%) [95%CI]	273 (74.4) [70.0–79.0]^a^	39 (84.8) [71.9–96.9]	0.146	58 (82.9) [74.3–91.4]	39 (84.8) [71.9–96.9]	0.805
1-year-mortality *n* (%) [95%CI]	141 (38.4) [33.8–43.6]	17 (53.1) [37.5–71.9]	0.073	37 (52.9) [40.0–64.3]	17 (53.1) [37.5–68.8]	0.989
BCLC B	*n* = 29	*n* = 24		*n* = 49	*n* = 24	
Target lesions	299	23		49	23	
Local tumor control *n* (%) [95%CI]	225 (75.3) [70.6–80.2]	19 (82.6) [66.7–95.8]	0.612	41 (83.7) [72.3–93.5]	19 (82.6) [66.7–95.8]	0.847
1-year-mortality *n* (%) [95%CI]	100 (33.4) [28.1–39.1]	11 [45.8) [30.0–71.4]	0.120	20 (40.8) [26.9–55.0]	11 (45.8) [29.2–70.8]	0.616
BCLC C	*n* = 68	*n* = 11		*n* = 21	*n* = 11	
Target lesions	68	23		21	23	
Local tumor control *n* (%) [95%CI]	48 (70.6) [60.0–81.0]	20 (87.0) [72.2–100]	0.272	17 (81.0) [64.0–95.8]	20 (87.0) [72.2–100]	0.648
1-year-mortality *n* (%) [95%CI]	41 (60.3) [48.3–70.9]	6 (5.4) [27.3–90.0]	0.999	17 (81.0) [64.0–95.8]	6 (5.4) [27.3–90.0]	0.397

^a^95%CI refers to the relative percentages

^b^Local tumor control refers to the treated target lesions

### Local tumor control and 1-year-mortality in patients with BCLC B and BCLC C

We further assessed LC at 1 year and 1-year-mortality in patients in BCLC stage B and C (Table 3). In the matched cohort LC was comparable in BCLC B patients treated with TACE compared to SBRT patients (83.7% vs. 82.6%, *p* = 0.847). In patients with BCLC C LC was higher in patients by trend higher in patients treated with SBRT compared to TACE patients (87.0% vs. 81.0%), but without reaching statistical significance (*p* = 0.648). 1-year mortality was similar in patients with BCLC B, however in BCLC C patients there was a trend to a higher 1-year mortality in patients treated with TACE (81.0% vs. 54.5%, *p* = 0.397, Table 3).

## Discussion

SBRT is currently not included in the HCC treatment algorithm of the current European guidelines [1, 3, 16]. However, there is growing evidence that SBRT can achieve good local tumor control in patients with HCC, even in patients with advanced liver disease with acceptable toxicity [6, 17]. Furthermore, SBRT as a bridging treatment to liver transplantation showed promising results and can be used as an alternative to conventional bridging treatments [2, 3, 8, 11]. Wahl et al. showed that SBRT was equally effective compared to radiofrequency ablation [18]. Since many patients are diagnosed with intermediate or even advanced stages HCC, it is therefore important to evaluate the role of SBRT in this clinical setting. In patients with intermediate HCC, TACE is the treatment of choice [19]. Importantly, many patients are treated with several TACE sessions to achieve a good local tumor control and in some patients further transarterial approaches may be limited due to impaired vascular architecture after several embolization procedures. In these patients sorafenib is standardly used by applying the concept of treatment stage migration. However, sorafenib is associated with several adverse events such as diarrhea and hand-foot syndrome which may limit treatment duration and therefore efficacy [20]. With regard to these adverse events which significantly reduce quality of life, SBRT may be a well-tolerated treatment [21–23]. Importantly, as shown in our unmatched cohort, patients treated with SBRT often present with advanced tumor stages. Therefore, SBRT patients had larger tumors and more often portal vein thrombosis (Table 1). In summary, there are significant differences in baseline characteristics in patients who are allocated to TACE or SBRT for HCC treatment. Being aware of these differences, we performed propensity score matching in order to adjust for these parameters which may be important for the analyzed outcome. However, as 98.6% of the patients treated with TACE had no prior HCC treatment and 83.0% of the SBRT patients had been previously been treated for HCC, we were not able to adjust for this variable as the differences were too large and sample size of the SBRT patient was too small.

After propensity score matching, we analyzed LC at 1 year after TACE or SBRT. The LC of 84.8% in SBRT patients was comparable to the LC of 82.9% in TACE patients (*p* = 0.805). Moreover, our LC at 1 year after SBRT was comparable to those reported in previous studies [6]. Further, we set out to determine the OS in our patients treated with TACE or SBRT. In the unmatched cohort, patients with TACE had significantly better OS compared to patients treated with SBRT (17.0 [14.4–19.6] months vs. 9.0 [6.7–11.3] months, *p* = 0.016) which may be explained by the significantly different baseline characteristics as they are well-known strong prognostic factors. However, after adjusting for these confounders, OS in patients with SBRT was similar to those of patients treated with TACE (11.0 [5.9–16.1] months in TACE patients vs. 9.0 [6.7–11.3] months in SBRT patients, *p* = 0.492). In accordance with the OS, the 1-year mortality rate in patients treated with SBRT was comparable to TACE patients (52.9% vs. 53.1%, *p* = 0.989). Our sub-group analyses in the matched cohort showed a trend to a higher 1-year-mortality in BCLC C patients treated with TACE compared to SBRT while LC was by trend higher in SBRT treated patients. Although not being statistically significant, these results may be the rationale for preferring TACE in BCLC B patients if technical feasible while BCLC C patients may be allocated to SBRT treatment. However, this suggestion has to be verified in prospective trials, especially taking into account prior HCC treatment, failure to previous TACE and technical feasibility of recurrent TACE.

Moreover, we evaluated adverse events after TACE and SBRT treatment. In patients treated with TACE, symptoms of postembolization syndrome occurred which resolved during symptomatic treatment. In patients treated with SBRT, although having received prior HCC treatment, toxicities were also moderate in concordance to published literature [6, 24]. Furthermore, radiotherapy is a very well tolerated treatment in terms of quality of life with the only observed deficits being temporary worsening of appetite and fatigue [23]. Combining the good local tumor control and the few adverse events, SBRT may emerge as an effective and safe treatment in patients with intermediate HCC and also in selected patients with advanced HCC.

We have to acknowledge several limitations of our study. Our study was a retrospective, single-center observational study with a limited sample size, especially of the SBRT patients. The decision for TACE or SBRT depended on several different factors such as intrahepatic tumor expansion, extent of PVT, liver function, the performance status of the patients and previous HCC therapies. We tried to reduce this bias by propensity score matching. However, matching was not perfect as we were not able to adjust for previous HCC therapies which would have resulted in a very small sample size without the possibility to perform statistical analyses. Therefore, prior HCC therapy may have affected outcome in patients with SBRT, especially as many of our SBRT patients had previous TACE treatment. However, according to the BCLC classification (TACE) is recommended as first-line treatment in patients with intermediate HCC. Only if TACE is technically not feasible or if contraindications do not allow to perform TACE, these patients may be allocated to SBRT treatment after multidisciplinary discussion. In summary, in everyday clinical practice, SBRT is currently not used as first-line treatment in these patients and therefore, these patients have received more prior HCC treatment compared to TACE patients so that this scenario represents everyday clinical practice. By considering this drawback, our results may indicate that patients who are treated with SBRT after prior HCC treatment including TACE have similar LC compared to patients who are only treated with TACE.

## Conclusion

Nevertheless, our results may be the rational for designing prospective, randomized-controlled trials to analyze the efficacy of SBRT compared to TACE. With these preliminary results in mind, we have already started a prospective, single-center study comparing TACE and SBRT in this clinical setting (HERAKLES, DRKS number: DRKS00008566) in order to determine the role of SBRT in the treatment algorithm of HCC.

## Additional file

Additional file 1: **Table S1.** Multivariate logistic regression model for propensity score matching. **Figure S1.** Standardized differences in the unmatched (black points) and matched cohort (redpoints). (DOCX 118 kb)

## Abbreviations

95%CI: 95% confidence interval
AFP: Alpha-fetoprotein
*ALT*: *Alanine aminotransferase*
*AST*: *Aspartat aminotransferase*
BCLC: Barcelona Clinic Liver Cancer
BED: Biological effective doses
CBCT: Cone beam computed tomography
*CT*: *Computerized tomography*
*cTACE*: *Conventional transarterial chemoembolization*
CTC: Common toxicity criteria
Dmax: Maximum dose
*EASL*: *European Association for The Study of Liver Diseases*
ECOG: *Eastern Cooperative Oncology Group*
EQD2: Equieffective doses for 2 Gy fractions
*GTV*: *Gross tumor volume*
Gy: Gray
HCC: Hepatocellular carcinoma
HR: Hazard ratio
IMRT: Intensity modulated radiotherapy
IQR: Interquartile range
*ITV*: Internal target volume
*LC*: *Local tumor control*
*MRI*: *Magnetic resonance imaging*
NAFLD: Non-alcoholic fatty liver disease
OAR: Organ at risk
OR: Odds ratio
*OS*: *Overall survival*
PVT: Portal vein thrombosis
RILD: Radiation induced liver disease
SBRT: Stereotactic body radiation therapy
SIP: Simultaneous integrated protection
TACE: Transarterial chemoembolization
β: Regression coefficient
