# Supplementary material for: Comparison of local tumor control in patients with HCC treated with SBRT or TACE: a propensity score analysis
Source: BMC Cancer. 2018 Aug 9;18:807. doi: 10.1186/s12885-018-4696-8 (PMC6085616; doi:10.1186/s12885-018-4696-8)
Supplement: Supplementary file 1 — Table S1. Multivariate logistic regression model for propensity score matching. Figure S1. Standardized differences in the unmatched (black points) and matched cohort (redpoints). (DOCX 118 kb) [file 12885_2018_4696_MOESM1_ESM.docx]

**Additional file 1**

**Table S1. Multivariate logistic regression model for propensity score matching.** OR represents the risk for being treated with SBRT.

| Variable | multivariate logistic regression model | | |  |
| --- | --- | --- | --- | --- |
|  | **β**^1^ | **OR**^2^ | **95%CI**^3^ | **p value** |
| segmental PVT^4^ | 0.27 | 1.31 | 0.56 - 3.04 | 0.533 |
| hepatic tumor expansion  (olignodular vs. multifocal) | 1.44 | 4.24 | 1.63 - 10.99 | 0.003 |
| largest tumor diameter | 0.17 | 1.12 | 1.08 - 1.30 | <0.001 |
| Child score | 0.28 | 1.32 | 1.01 - 1.74 | 0.044 |
| viral liver disease | -1.02 | 0.36 | 0.12 - 1.08 | 0.067 |
| ECOG^5^ 0 vs. 1/2 | 0.30 | 1.36 | 0.59 - 3.13 | 0.476 |

Abbreviations: ^1^β, regression coefficient; ^2^OR, Odds ratio; ^3^95%CI, 95% confidence interval; ^4^PVT, portal vein thrombosis; ^5^ECOG, Eastern Cooperative Oncology Group

**Figure S1. Standardized differences in the unmatched (black points) and matched cohort (red points).**
